# Optimizing resistance training for pain management in knee and hip osteoarthritis: a pairwise and dose–response meta-analysis

**DOI:** 10.3389/fpubh.2025.1623679

**Published:** 2025-09-02

**Authors:** Wenyu Liu, Meng Yin, Huimin Li

**Affiliations:** ^1^School of Physical Education, Hubei University of Automotive Technology, Shiyan, China; ^2^Department of Sport, Dongshin University, Naju, Jeollanam-do, Republic of Korea; ^3^Yantai Gold College, Yantai, China

**Keywords:** osteoarthritis, resistance training, pain, meta-analysis, dose–response

## Abstract

**Background:**

Osteoarthritis (OA) is a degenerative joint disease affecting approximately 300 million people worldwide. OA manifests as significant pain and stiffness as well as reduced mobility, substantially impacting patient quality of life and imposing considerable financial burdens on healthcare systems. Although resistance training (RT) demonstrates therapeutic potential, existing studies vary widely in its intensity, duration, and effectiveness, necessitating comprehensive dose–response analyses.

**Objective:**

This study aimed to evaluate the effectiveness of RT interventions in the management of OA pain.

**Methods:**

A systematic literature search was performed of the PubMed/MEDLINE, Embase, Cochrane Library, and Web of Science databases. Effect sizes were computed using Hedges’g, while the risk of bias was assessed using the Cochrane Risk of Bias 2 tool. Potential moderating factors including age, sex, and body mass index (BMI) were also analyzed.

**Results:**

The analysis included 28 randomized controlled trials (2,164 participants) that satisfied the inclusion criteria. RT significantly reduced OA pain compared to no intervention (Hedges’g = −0.57; 95% CrI, −0.65 to −0.49). A U-shaped dose–response relationship was observed, with an optimal weekly RT dose of 680 METs/min/week for pain relief. Higher or lower doses were less effective, and pain improvement was maintained for up to 6 months post-intervention. Age and sex were potential moderators, with more significant benefits observed in females and less favorable outcomes in older patients. BMI had no significant effect on RT efficacy.

**Conclusion:**

RT constitutes an effective non-pharmacological intervention for reducing OA pain, at an optimal training dose of 680 METs/min/week. These findings emphasize the importance of considering individual patient characteristics, particularly age and sex, when prescribing RT for OA pain management.

**Systematic review registration:**

PROSPERO, Identifier: CRD42024622698; https://www.crd.york.ac.uk/PROSPERO/view/CRD42024622698.

## Introduction

Osteoarthritis (OA) is among the most prevalent degenerative joint diseases worldwide (1). The World Health Organization estimates that approximately 300 million people worldwide are affected by OA, with prevalence rates reaching 10% in adults aged ≥ 65 years (2). Among the various types of OA, knee and hip OA are the most common, significantly contributing to disability and reduced quality of life in affected individuals (3). The primary clinical manifestations of OA include pain, stiffness, reduced range of motion, and muscle weakness, which subsequently lead to functional limitations (4). The substantial impact of osteoarthritis (OA) on quality of life translates into a major economic burden on health-care systems, costing about USD 137 billion each year in the United States and absorbing 1–2.5% of gross national product in other high-income economies (5). Current standard treatments, while utilized, have notable limitations. Pharmacological interventions like nonsteroidal anti-inflammatory drugs are associated with significant gastrointestinal and cardiovascular risks (6). Although arthroplasty is an option for severe cases, it is an invasive procedure with considerable risks and a demanding rehabilitation period (7). These shortcomings underscore the urgent need for safe and effective non-pharmacological alternatives, making the optimization of interventions like resistance training (RT) a clinical priority.

Muscle weakness affects OA progression and is strongly linked to pain, functional limitations, and the risk of falling. Studies have shown that reduced muscle strength may be a risk factor for disease progression; therefore, optimizing muscle strength plays a crucial role in its prevention (8). Resistance training (RT), a standard strength training method, is an important treatment for OA (9). Behnam et al. demonstrated that RT alleviates pain and improves knee stability by enhancing muscle strength, thereby reducing joint loading and slowing OA progression (10).

However, although existing studies have shown that RT can relieve OA symptoms, interstudy variations in its intensity, frequency, and duration have created uncertainty in efficacy assessments and prevented the determination of an optimal intervention program (11, 12). Existing meta-analyses have limited ability to evaluate the effectiveness of RT in patients with OA. Many meta-analyses focused solely on pairwise comparative analyses and failed to comprehensively assess the benefits of RT by incorporating the relative efficacy of multiple treatment modalities (4, 13). While previous meta-analyses have confirmed that RT is beneficial for OA patients, they have been largely dose-agnostic. These studies typically treated RT as a monolithic intervention, failing to disentangle the critical influence of its dose, namely the intensity, frequency, and duration of the exercise. This has created a significant gap in clinical knowledge, as the question is no longer if RT is effective, but what dose of RT is optimal for pain management. By focusing only on whether RT works, prior work has provided limited guidance for prescribing specific, optimized exercise protocols (14). Our study aims to address this precise gap by conducting a comprehensive dose–response meta-analysis. From a public health perspective, this gap is particularly critical. The absence of clear, evidence-based dosage guidelines hinders the development and implementation of scalable, cost-effective exercise programs for managing OA within aging populations. Therefore, determining an optimal dose moves beyond individual clinical prescription and becomes essential for informing public health policy, enabling community-level interventions, and ultimately reducing the societal burden of OA.

Given these limitations, this study aims to address these gaps through a systematic review and dose–response meta-analysis. Specifically, our primary objectives were to: (1) determine the overall effectiveness of resistance training (RT) compared to non-exercise controls for pain reduction in adults with knee or hip osteoarthritis (OA); and (2) investigate the dose–response relationship between weekly RT volume (measured in METs-min/week) and pain improvement to identify an optimal dosage range. We also explored potential moderators of the treatment effect, including age, sex, and body mass index (BMI).

## Methods

This systematic review and meta-analysis was conducted and reported in accordance with the Preferred Reporting Items for Systematic Reviews and Meta-analyses (PRISMA) 2020 statement (15).

### Registration

This meta-analysis adhered to the guidelines of the Cochrane Handbook for Systematic Reviews of Interventions (16). This study was registered on the PROSPERO prospective registry platform (CRD42024622698).

### Search strategy

The MEDLINE (via PubMed), Embase, Cochrane Library, and Web of Science databases were systematically searched for relevant studies published from inception through December 2024. The search strategy used a combination of Medical Subject Headings (MeSH) terms and free words, with the primary MeSH terms including: “Osteoarthritis, Hip,” “Osteoarthritis, Knee,” and “Resistance Training.” No restrictions were placed on language or region of publication. We also manually searched the reference lists of the retrieved articles to prevent omissions. Supplementary file 1 provides the complete search strategy for each database.

### Inclusion criteria and exclusion criteria

Studies were included based on the following criteria, structured according to the Population, Intervention, Comparator, Outcomes, and Study Design (PICOS) framework:

Population: Adults (aged ≥18 years) with a clinical or radiographic diagnosis of knee or hip osteoarthritis (17).

Intervention: The intervention group received a resistance training (RT) program designed to improve muscle strength and endurance. This included exercises using external forces (e.g., weights, elastic bands) or the participant’s own body weight (18).

Comparator: The control group received a non-exercise intervention, such as usual care, health education, or placement on a waiting list.

Study Design: Only randomized controlled trials (RCTs) were included.

Studies were excluded if they only reported on the acute (single-session) effects of RT.

### Study selection

Two independent reviewers screened the titles, abstracts, and texts of the included studies according to the eligibility criteria. The main tool used was Endnote X9 (Clarivate, Philadelphia, PA, United States). When the two reviewers disagreed, a third experienced reviewer adjudicated.

### Data extraction and management

Two reviewers worked independently to extract data from all included studies using a standardized data extraction form designed for this review. Any disagreements were resolved through discussion or, if necessary, consultation with a third reviewer. The following information was extracted from each study:

Study Characteristics: Author, year of publication, country, and funding source.

Participant Characteristics: Sample size, mean age, percentage of female participants, mean BMI, type of osteoarthritis (knee or hip), and duration of illness.

Intervention Details: For the RT group, we extracted the total duration of the intervention (in weeks), exercise frequency, session duration, prescribed intensity (METs), supervision status (yes/no), and the calculated weekly exercise dose (METs-min/week). For example, if high-intensity RT expends 6.5 metabolic equivalents per minute (METs/min) and is performed three times per week for 60 min each, the weekly burn would be 6.5 × 3 × 60 = 1,170 METs/min. RT intensities were compared to those in the physical activity expenditure in the 2024 Adult Compendium of Physical Activities publication (19).

Comparator Details: The type of control intervention (e.g., usual care, health education, waiting list).

Outcome Data: For the primary outcome of pain, we extracted the mean, standard deviation (SD), and sample size for both intervention and control groups at baseline and post-intervention timepoints.

### Risk of bias and evidence quality

Two reviewers independently assessed the risk of bias for each included study. As all included studies were RCTs, we used the Cochrane Risk of Bias 2 (RoB 2) tool (20). Any disagreements between reviewers were resolved through discussion or consultation with a third reviewer. The RoB 2 tool involves a domain-based evaluation, assessing bias across five areas: bias arising from the randomization process, bias due to deviations from intended interventions, bias due to missing outcome data, bias in measurement of the outcome, and bias in selection of the reported result. For each domain, a judgment of “Low risk,” “Some concerns,” or “High risk” was assigned based on signaling questions outlined in the RoB 2 guidance. An overall risk of bias judgment for each study was then determined: a study was rated as “Low risk” only if all domains were assessed as low risk; “High risk” if at least one domain was assessed as high risk, or if multiple domains had “Some concerns”; and “Some concerns” for all other situations.

### Measures of treatment effect

Effect sizes were estimated by Hedges’g due to its ability to adjust for studies with small sample sizes, thus reducing bias. Hedges’g is a modified form of the standardized mean difference, which is commonly used to compare effect sizes between groups, particularly among studies using different measurement scales (21). Hedges’g was calculated from the mean and standard deviation of pre- and post-intervention values. If standard deviations were missing, standard errors, 95% confidence intervals, or t-statistics were used to calculate SD (16). Finally, we assumed a correlation coefficient of *r* = 0.5 between the pre- and postintervention measurements to reflect the moderate correlation between pre- and post-intervention changes (16).

### Statistical analysis

The pairwise meta-analysis was conducted using the “brms” package in R (version 4.3.1), with Bayesian random effects modeling to estimate effect sizes for RT. The “brms” package performs Bayesian modeling based on the probabilistic programming language Stan, allowing flexibility in managing complex model structures and posterior inference via Markov Chain Monte Carlo methods (22). We employed a weakly informative prior for the intercept parameter (prior distribution of the overall effect size *μ* [0, 1], interstudy heterogeneity Tau [0, 1]) (23). To ensure convergence of the Bayesian model, we used the potential scale reduction factor to evaluate the convergence of each parameter, with a value of <1.05 indicating good model fit (24). Heterogeneity was measured using SD in the model to reflect the degree of variation in the effect sizes of individual studies (25). We used 95% credible intervals (CrIs) to assess the estimated uncertainty of the RT effect. We used “ggplot2” to visualize all analyses. Finally, we conducted Egger’s and Begg’s regression tests and plotted funnel plots to examine potential publication bias (26).

We conducted a regression analysis to examine possible moderators. The variables of interest included weekly RT doses, number of weeks of intervention, number of weeks of follow-up, age, sex, and BMI. To model the potentially non-linear relationship between RT dose and pain improvement, we utilized a natural spline-based model with 4 knots (27). In simple terms, splines are a flexible statistical tool that fits a series of smooth, connected polynomial curves to different segments of the data. This allows the model to capture complex patterns, such as the U-shaped relationship observed in our study, without being forced into a predefined linear or quadratic shape. We also conducted linear regression analyses of the participants’ characteristics. Furthermore, we conducted a subgroup analysis to investigate whether unsupervised RT affected the results.

To facilitate the interpretation of the findings and improve their generalizability, we assessed the minimum clinically important difference (MCID) in pain improvement in patients with OA in terms of effect size and dose range. As described by Simon et al. (28), we preset the MCID to 0.37 SD units, the smallest median clinically important difference found in studies of patients with OA. We then predicted the dose of the RT modality required to achieve the combined effect size of the MCID.

## Results

### Study selection

**Figure 1:**
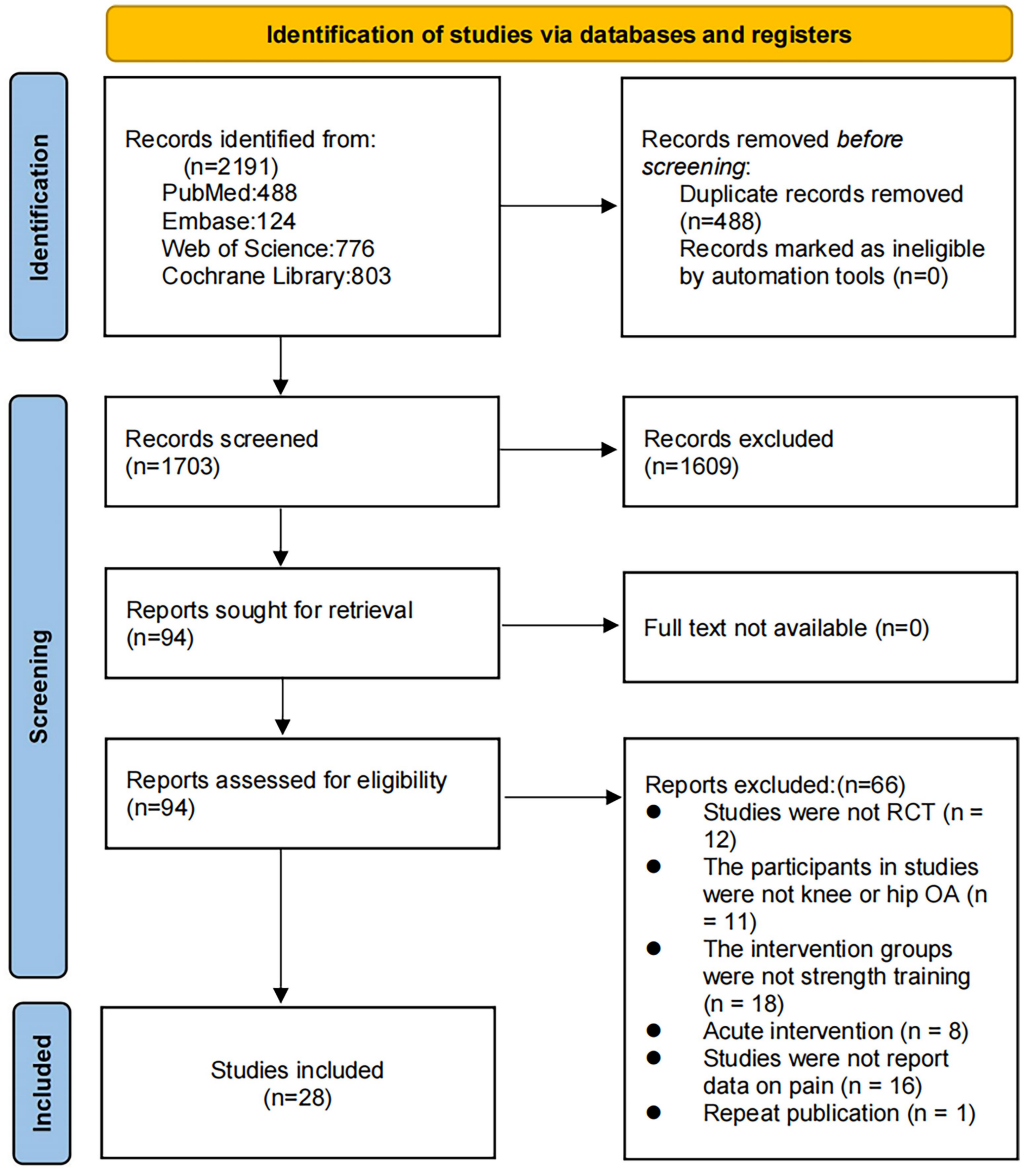
Literature review flowchart.

We retrieved 2,191 articles through this database review. After the removal of 488 duplicates, 1,703 articles were subjected to title and abstract review, which eliminated 1,609 of them. Thus, 94 articles were subjected to the full-text evaluation. Of them, 66 failed to meet the inclusion criteria (i.e., were non-RCTs, did not involve patients with OA, included intervention groups other than RT, focused on acute interventions, failed to report pain-related data, or were duplicate publications). Finally, 28 studies met the inclusion criteria (Figure 1).

### Study characteristics

The 28 RCTs included 2,164 participants (1,259 in the RT group). The mean patient age ranged from 54.8 to 86.1 years, while the mean BMI ranged from 24.6 to 34.7 kg/m^2^. The mean intensity per minute of RT ranged from 3.5 to 7 METs/min, was performed a mean one to five times per week, had a mean duration that ranged from 4 to 72 weeks, and had a mean follow-up period of 12 to 96 weeks. Further details about study inclusion are shown in Supplementary file 2.

### Risk of bias and evidence quality

Supplementary file 3 presents the risk of bias of the included studies. Of the 28 studies, five were categorized as having a high risk of bias, four as having a low risk of bias, and 19 as demonstrating “some concerns.” Owing to the risk of bias and inconsistency, the quality of evidence in this study was rated as low (Table 1).

**Table 1:** Pairwise meta-analysis for all outcomes.

SMD	Lower 95% CrI	Upper 95% CrI	PSRF	SD (Intercept)	SD-lower 95% CrI	SD-upper 95% CrI	GRADE	Bulk_ESS	Tail_ESS
−0.57	−0.65	−0.49	1.00	0.29	0.21	0.40	Low^1,2^	31,367	18,569

SMD, standard mean difference. SE, standard error. CrI, credible interval. SD, standard deviation. 1: Risk of bias. 2: Inconsistency. Bulk_ESS, Bulk Effective Sample Size: indicates the effective sample size of the Markov Chain Monte Carlo (MCMC) samples, which primarily assesses the mixing of the chains within the main distribution region. Tail_ESS (Tail Effective Sample Size): evaluates the effective sample size of Markov chain Monte Carlo samples in the tail (extreme value) region of the parameter distribution.

### Pairwise meta-analysis

**Figure 2:**
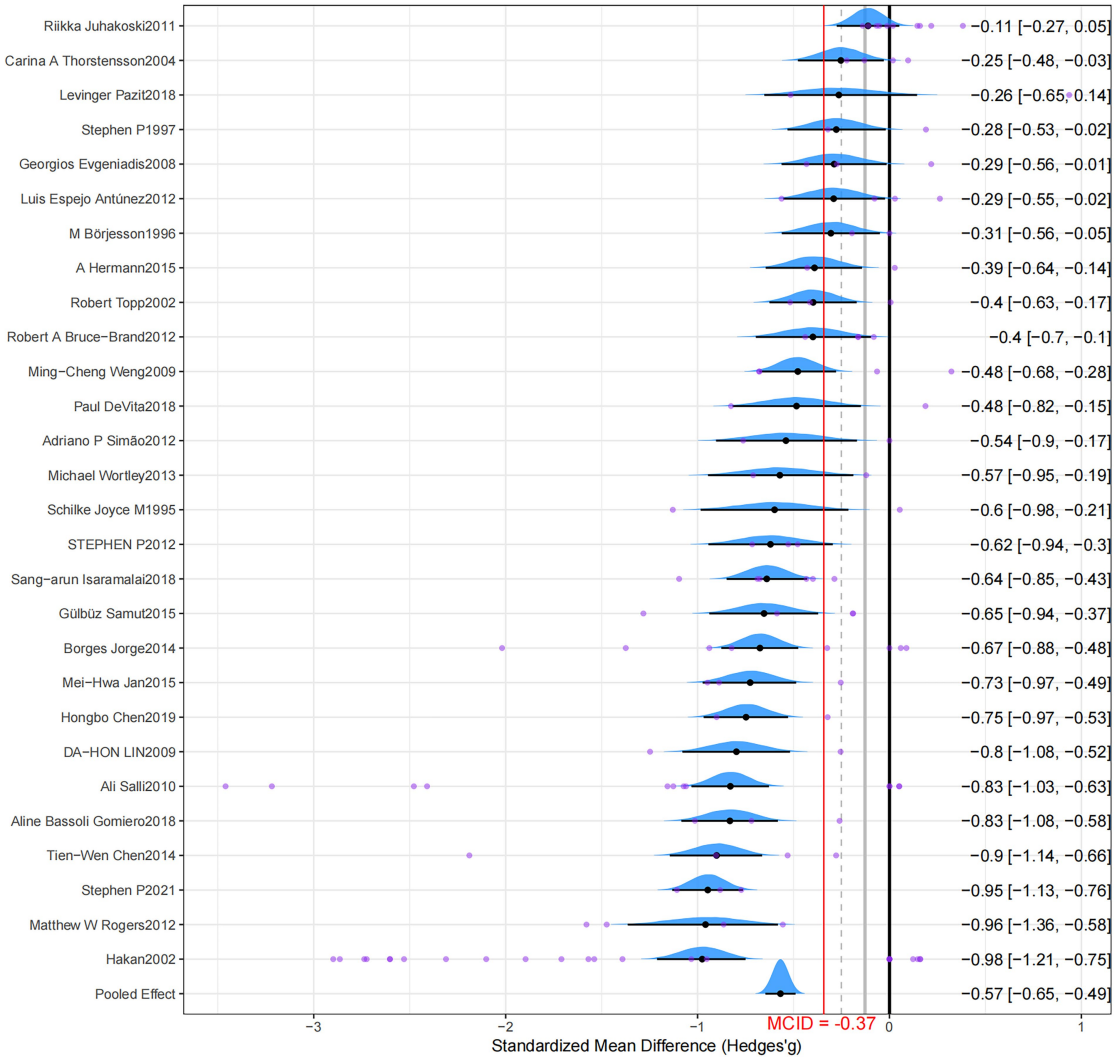
Forest plot.

The pairwise analyses and forest plots are shown in Figure 2 and Table 1, respectively. The forest plot visually summarizes the results, displaying the effect size and 95% credible interval for each individual study alongside the overall pooled effect, which demonstrates a consistent trend of benefit from RT across most studies. Compared to a non-exercise control group, RT significantly reduced pain in patients with OA, and a moderate effect exceeded the MICD (Hedges’g = −0.57; 95% CrI, −0.65 to −0.49; grade: low). Moderate heterogeneity was observed in the results, and the model exhibited good fit (SD = 0.29; 95% CrI, 0.21–0.40; RSPF = 1.00). After correction, the funnel plot exhibited asymmetry and significance for both Egger’s and Begg’s tests (*p* < 0.001), suggesting potential publication bias (Supplementary file 4).

### Dose–response meta-analysis

**Figure 3:**
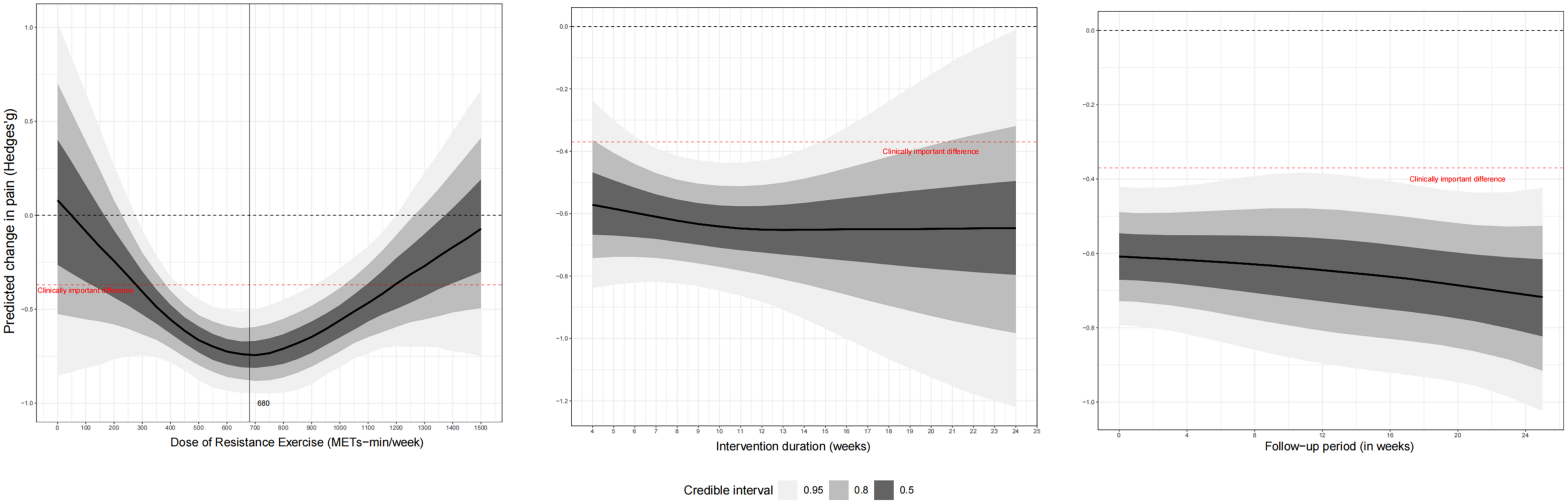
Dose–response analysis.

Figure 3 shows the dose–response results of the RT. The spline model revealed a distinct U-shaped (or inverted U-shaped) non-linear relationship between the weekly RT dose and pain reduction, indicating that benefits increase with dose up to an optimal point, after which higher doses lead to diminished effects. We observed a U-shaped nonlinear dose–response relationship between weekly RT doses and pain outcomes. The MCID was reached when the weekly training dose was 280 METs/min (Hedges’g = −0.36; 95% CrI, −0.67 to −0.05) and optimal weekly training dose was 680 METs/min (Hedges’g = −0.73; 95% CrI, −0.91 to −0.56), while the maximum tolerated dose was reached when the weekly dose exceeded 1,180 METs/min (Hedges’g = −0.37; 95% CrI, −0.64 to −0.09). In addition, changes in the total intervention time did not significantly affect the intervention’s effect, and the RT was able to alleviate pain for 6 months.

### Other moderating factors

**Figure 4:**
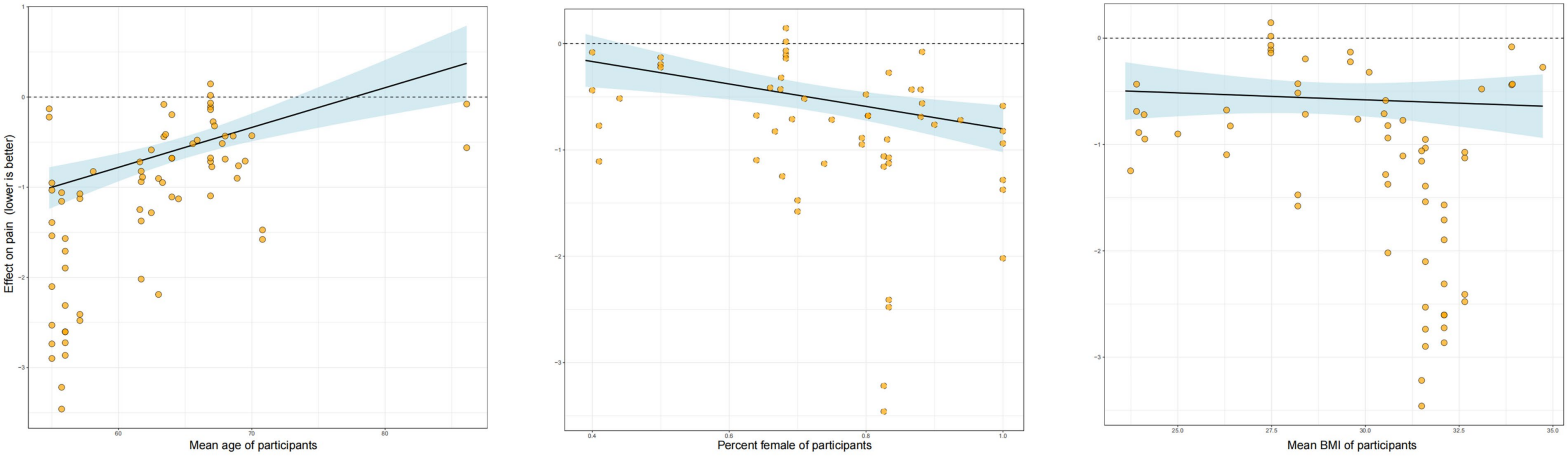
Meta-regression.

**Figure 5:**
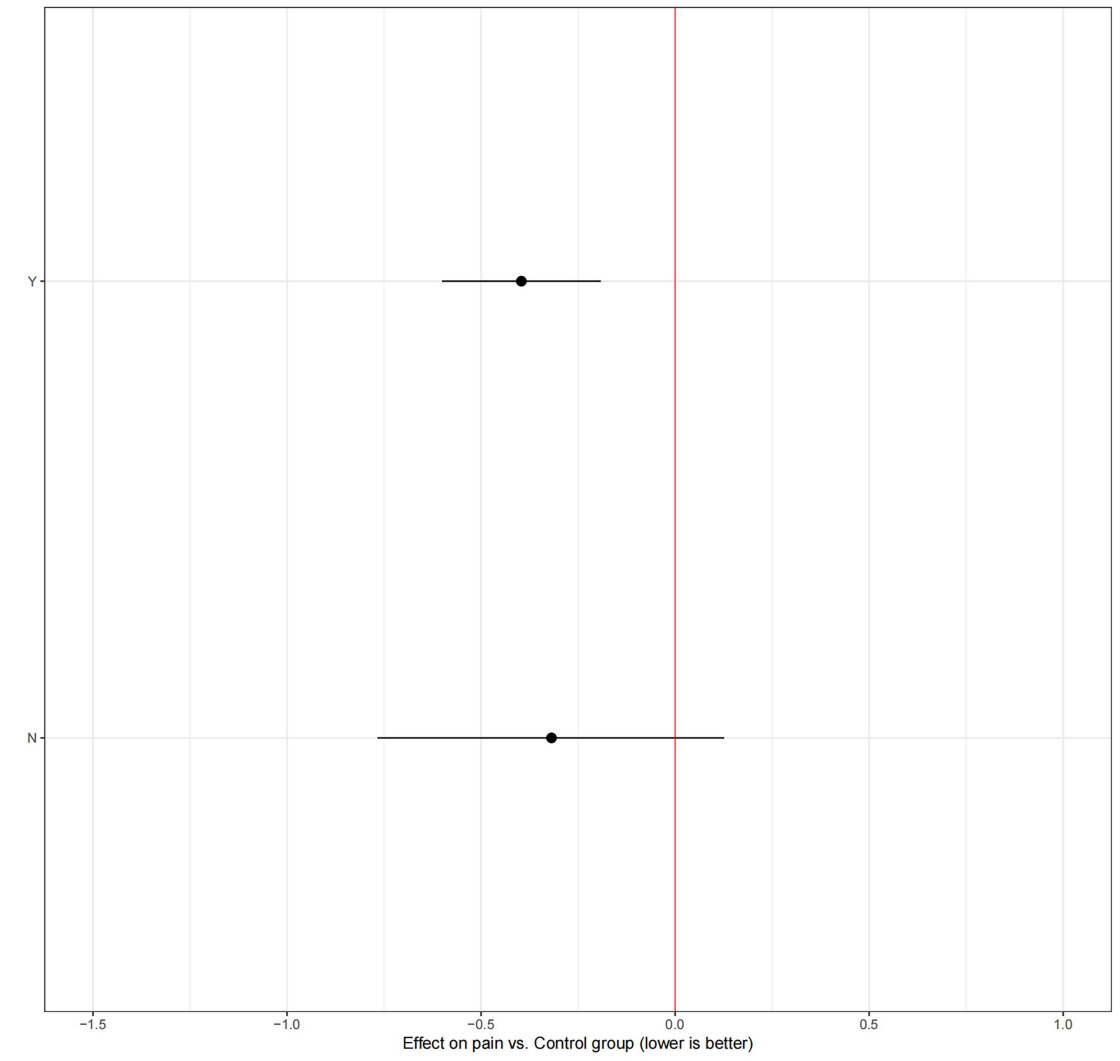
Subgroup analysis of supervision.

Our analysis of potential moderators revealed several key insights (Figure 4). Meta-regression indicated that age and sex significantly influenced the effectiveness of RT. Specifically, there was a negative association with age, suggesting that the pain-relieving benefits of RT were less pronounced in studies with older participants. Conversely, a higher proportion of female participants in a study was associated with a greater treatment effect. Baseline BMI, however, did not emerge as a significant moderator of the intervention’s effect. In a separate subgroup analysis comparing supervised versus unsupervised RT (Figure 5), the effect estimate for unsupervised RT was not statistically significant and was characterized by a wide credible interval, indicating considerable uncertainty and suggesting that supervised training may be more reliably effective. Additionally, the intervention weeks, follow-up duration, and supervision may improve the model’s fit (Appendix 6).

## Discussion

### Principal findings

In this meta-analysis of RCTs of OA, RT therapies were moderately to highly effective at reducing pain. The amount of RT showed a U-shaped relationship with pain improvement. A RT dose of 680 METs/min/week produced the best improvement in pain, while a RT dose of 300 METs/min/week produced the smallest MCID. The maximum RT dose was 1,180 METs/min/week. BMI did not hinder pain improvement after RT; however, the effect of RT on pain diminished with age. Regarding sex, female patients showed better improvement than male patients. A follow-up study showed that the improvement in OA pain persisted for 6 months after the cessation of RT. This finding is clinically meaningful as it suggests that a structured RT program can provide durable, medium-term relief, which is a key goal in the management of a chronic condition like OA.

### Comparison with other studies and future directions

Our results suggest that RT effectively improves the outcomes of patients with OA-related pain. A previous study found that improved knee extensor strength mediated the effect of a strengthening program on pain relief and physical function at 12 weeks (29). In a previously published meta-analysis by Filipe, RT performed for 1 h three to five times per week for 8–12 weeks improved knee flexor strength and power (12). Positive effects were also observed in knee extensor function, strength, and resistance (30). RT reduced pressure–pain sensitivity and pain thresholds, confirming that it is an effective non-pharmacological treatment for OA (31). Another study showed that RT significantly improved pain, muscle strength, maximum step speed, chair rise time, and balance among patients with OA. The studies indicated that 44% of current RT studies used machines for RT versus 44% using free weights. A gradual increase in RT load over 1–6 months was recommended by most studies (11).

The benefits of RT on OA pain are likely multifactorial. On a biomechanical level, strengthening the muscles surrounding the knee and hip, such as the quadriceps, improves joint stability and enhances shock absorption, thereby reducing the load on the articular cartilage (32, 33). This can interrupt the cycle of joint degradation and pain. Furthermore, emerging evidence points to systemic physiological benefits. Studies have shown that structured exercise can exert an anti-inflammatory effect, potentially by reducing pro-inflammatory cytokines like TNF-*α* and IL-6, which are implicated in OA pathogenesis and pain sensitization (34, 35). Beyond the local joint, RT may also influence central pain processing. The concept of exercise-induced hypoalgesia suggests that physical activity can activate the body’s endogenous opioid and non-opioid analgesic pathways, effectively raising the pain threshold (36).

A particularly noteworthy finding of the current study is its determination of the optimal RT dose. This study demonstrated a U-shaped relationship between RT dose and pain improvement. However, neither higher nor lower RT doses achieved optimal pain relief. This finding supports previous findings that a low exercise dose may not result in significant physiological changes. One hypothesis for this downturn at higher doses is that excessive training volumes may lead to joint irritation or overtraining, potentially counteracting the benefits (37). However, as this was not directly measured, this remains a speculative point requiring further investigation. Nevertheless, higher training doses may improve knee function and pain in older patients with OA (38), indicating that training doses may have varied effects across different populations. Here we provided recommended dosages for six different types of RT, including the energy expenditure (in METs/min) of each session, recommended total weekly time, and specific training schedule, detailing the duration of each workout and number of workouts per week (Table 2) (19).

**Table 2:** Resistance dose recommendation.

Type of resistance training	Code^1^	Energy expenditure (METs-min)	Recommended accumulation^2^ (min/ week)	Energy expenditure^3^ (METs-min)
Resistance (weight lifting - free weight, nautilus or universal-type), power lifting or body building, vigorous effort	02050	6.0	113	60 × 2
				120 × 1
Resistance (weight) training, squats, deadlift, slow or explosive effort	02052	5.0	136	65 × 2
				45 × 3
Resistance (weight) training, multiple exercises, 8–15 reps at varied resistance	02054	3.5	194	65 × 3
				50 × 4
Resistance Training, circuit, reciprocol supersets, peripheral hear action training	02055	5.8	117	60 × 2
				120 × 1
Body weight resistance exercises (e.g., squat, lunge, push-up, crunch), general	02056	3.0	226	55 × 4
				75 × 3
Body weight resistance exercises (e.g., squat, lunge, push-up, crunch), high intensity	02057	6.5	104	50 × 2
				100 × 1

1 The codes are from the 2024 Adult Compendium of Physical Activities (1).

2 Recommended optimal weekly dose.

3 Exercise time does not include warm-up and relaxation (1).

Unlike previous studies, our study found that the interventional duration was not a significant factor in determining the effect of RT on pain improvement among patients with OA. Similar to a previous meta-analysis, we found that most RT durations were 1–30 months in duration (11). Furthermore, in our study, BMI did not influence the effect of RT on pain improvement, suggesting that RT can strengthen the knee joint and surrounding muscle groups irrespective of a patient’s weight (39). This finding is consistent with that of a previous study that reported that a 6-week isokinetic knee RT program improved the muscle strength, joint range of motion, physical performance, pain, and quality of life of overweight/obese women with bilateral patellofemoral pain syndrome. Notably, the pain-relieving effect of RT decreases with age, possibly because of a diminished exercise effect resulting from reduced physical function and recovery capacity (40). Our finding that female patients experienced greater pain relief is particularly intriguing, especially given that women often report higher pain severity for the same degree of radiographic OA (41). The underlying mechanisms for this enhanced response to RT are complex. Hormonal differences may play a role, as estrogen is known to have potential chondroprotective (cartilage-protective) effects, and its fluctuation could influence both joint health and pain perception (42). From a biomechanical standpoint, women typically have different pelvic anatomy and a wider Q-angle, which can alter joint loading mechanics. It is plausible that RT is particularly effective at correcting or compensating for these baseline biomechanical challenges (43). Finally, there are known sex differences in central pain processing pathways, and it is possible that RT interacts with these pathways differently in females, leading to a more pronounced analgesic effect (44). The observation that the benefits of RT diminished with increasing age aligns with our understanding of age-related physiological changes. Older adults often exhibit a phenomenon known as “anabolic resistance,” where the muscle’s protein synthesis response to a given stimulus (like exercise or nutrition) is blunted compared to younger individuals (45). This could mean that for the same relative training dose, older patients may experience smaller gains in muscle mass and strength, leading to a less pronounced effect on joint stability and pain. Additionally, aging is associated with a baseline pro-inflammatory state (“inflammaging”) and potentially slower tissue repair and recovery processes (46). Therefore, while RT is still beneficial, its overall efficacy might be attenuated in older individuals due to these underlying biological constraints.

### Clinical implications and practical guidance

This study is the first Bayesian multilevel meta-analysis to investigate the effect of RT on pain in patients with knee and hip OA. It clarifies the effectiveness of RT in relieving pain and the potential influence of different variables. In addition, we have recommended exercises based on the optimal RT dose to help doctors or patients customize an individualized exercise prescription (Table 2). Crucially, the practical guidance derived from our findings extends into the realm of public health. The identification of an optimal dose (approximately 680 METs/min/week) and the concrete examples provided in Table 2 can serve as a scientific foundation for community health organizations and public health bodies. It enables them to design and promote standardized, scalable exercise interventions aimed at reducing the burden of OA at the population level, thereby promoting active aging and potentially reducing healthcare costs. However, patient-specific factors should also be considered, and clinicians should thoroughly assess each patient’s needs, preferences, and physical abilities before prescribing RT therapy. Furthermore, the need for supervision and potential resource constraints should be considered, particularly as our findings suggest supervised RT is more reliably effective. Supervision can enhance adherence, ensure proper exercise form to prevent injury, and facilitate appropriate progressive overload, which are all critical for optimal outcomes. Although the confidence in these results was rated as low to very low, our findings provide valuable insight into optimizing specific RT therapies for patients with OA.

### Strengths

The principal strength of this study is its innovative methodology, which pioneered the use of a Bayesian modeling based on a multilevel meta-analysis to investigate the effects of RT in patients with OA. This approach facilitated comprehensive data synthesis through specific features that enabled the integration of effect sizes from different studies. Previous meta-analyses of exercise interventions demonstrated the robustness and validity of this methodological approach. The implementation of the “brms” Bayesian package enables a comprehensive assessment of the participants’ baseline characteristics, study details, and effect of exercise dose on outcomes, thereby enabling a flexible analysis of complex data. The examination of RT modalities provides new insights into optimal treatment strategies for OA.

### Limitations

Several limitations of this meta-analysis should be acknowledged. First, the overall quality of the included evidence was low, with only four of the 28 included trials judged as having a low risk of bias. This, combined with the moderate statistical heterogeneity observed in our main analysis (SD = 0.29), warrants caution when interpreting the pooled effect size. Second, our analysis detected a significant risk of publication bias, suggesting that smaller studies with null findings may be underrepresented, potentially leading to an overestimation of the treatment effect. Third, methodological limitations include the exclusion of the Scopus database from our search and the imprecision inherent in using standardized METs values to calculate exercise dose, which does not account for individual variability. Finally, the generalizability of our findings may be limited. The lack of follow-up data beyond 1 year prevents conclusions about the long-term sustainability of RT benefits, and our dose–response curve may not be applicable to patients with severe comorbidities or high frailty, who are often excluded from RCTs.

### Conclusion

This meta-analysis suggests that RT is a promising non-pharmacological intervention for managing pain in patients with knee and hip osteoarthritis, with an optimal dose identified at approximately 680 METs-min/week. However, these findings must be interpreted with considerable caution. The overall quality of the available evidence was rated as low, and our analysis detected significant publication bias, which implies that the true effect size may be smaller than what we have reported. Furthermore, the heterogeneity across included studies suggests that patient responses can be variable. Therefore, while our results provide a valuable framework for prescribing RT, clinicians should view the recommended dose as a guiding principle rather than a rigid rule. Treatment must be highly individualized, considering patient preferences, functional capacity, and comorbidities, with an emphasis on starting conservatively and progressing as tolerated. Future research should therefore focus not only on conducting more robust, large-scale trials to validate these dosage guidelines but also on investigating effective strategies for translating these findings into accessible and sustainable public health programs for aging populations.

## Data Availability

The original contributions presented in the study are included in the article/Supplementary material, further inquiries can be directed to the corresponding author.

## References

[1] Yamato TP Deveza LA Maher CG. Exercise for osteoarthritis of the knee (PEDro synthesis). Br J Sports Med. (2016) 50:1013–4. doi: 10.1136/bjsports-2016-096104, PMID: 27015856

[2] Glyn-Jones S Palmer AJ Agricola R Price AJ Vincent TL Weinans H . Osteoarthritis. Lancet. (2015) 386:376–87. doi: 10.1016/S0140-6736(14)60802-3, PMID: 25748615

[3] Cross M Smith E Hoy DG Nolte S Ackerman IN Fransen M . The global burden of hip and knee osteoarthritis: estimates from the global burden of disease 2010 study. Ann Rheum Dis. (2014) 73:1323–30. doi: 10.1136/annrheumdis-2013-204763, PMID: 24553908

[4] Ceballos-Laita L Lahuerta-Martín S Carrasco-Uribarren A Cabanillas-Barea S Hernández-Lázaro H Pérez-Guillén S . Strength training vs. aerobic training for managing pain and physical function in patients with knee osteoarthritis: a systematic review and Meta-analysis. Health. (2024) 12:33. doi: 10.3390/healthcare12010033, PMID: 38200939 PMC10778769

[5] Leifer VP Katz JN Losina E. The burden of OA-health services and economics. Osteoarthr Cartil. (2022) 30:10–6. doi: 10.1016/j.joca.2021.05.007, PMID: 34023527 PMC8605034

[6] Ikdahl E Kerola A Sollerud E Semb AG. Cardiovascular implications of non-steroidal anti-inflammatory drugs: a comprehensive review, with emphasis on patients with rheumatoid arthritis. European Cardiol. (2024) 19:e27. doi: 10.15420/ecr.2024.24, PMID: 39872418 PMC11770528

[7] Mori Y Tarasawa K Tanaka H Kamimura M Harada K Mori N . Thromboembolic and infectious complication risks in TKA and UKA: evidence from a Japanese nationwide cohort. Knee Surg Related Res. (2025) 37:19. doi: 10.1186/s43019-025-00273-6, PMID: 40341061 PMC12063263

[8] Lo GH Richard MJ McAlindon TE Kriska AM Price LL Rockette-Wagner B . Strength training is associated with less knee osteoarthritis: data from the osteoarthritis initiative. Arthritis Rheumatol (Hoboken, NJ). (2024) 76:377–83. doi: 10.1002/art.42732, PMID: 37870119 PMC10922276

[9] Guede-Rojas F Benavides-Villanueva A Salgado-Gonzalez S Mendoza C Arias-Alvarez G Soto-Martinez A . Effect of strength training on knee proprioception in patients with knee osteoarthritis: a systematic review and meta-analysis. Sports Med Health Sci. (2024) 6:101–10. doi: 10.1016/j.smhs.2023.10.005, PMID: 38708322 PMC11067762

[10] Liaghat B Bojsen-Møller J Juul-Kristensen B Henriksen P Mohammadnejad A Heiberg BD . High-load strength training compared with standard care treatment in young adults with joint hypermobility and knee pain: study protocol for a randomised controlled trial (the HIPEr-knee study). BMJ Open. (2024) 14:e090812. doi: 10.1136/bmjopen-2024-090812, PMID: 39414294 PMC11487976

[11] Lange AK Vanwanseele B Fiatarone Singh MA. Strength training for treatment of osteoarthritis of the knee: a systematic review. Arthritis Rheum. (2008) 59:1488–94. doi: 10.1002/art.24118, PMID: 18821647

[12] Raposo F Ramos M Lúcia Cruz A. Effects of exercise on knee osteoarthritis: a systematic review. Musculoskelet Care. (2021) 19:399–435. doi: 10.1002/msc.1538, PMID: 33666347

[13] Culvenor AG Ruhdorfer A Juhl C Eckstein F Øiestad BE. Knee extensor strength and risk of structural, symptomatic, and functional decline in knee osteoarthritis: a systematic review and Meta-analysis. Arthritis Care Res. (2017) 69:649–58. doi: 10.1002/acr.23005, PMID: 27563843

[14] Hua J Sun L Teng Y. Effects of high-intensity strength training in adults with knee osteoarthritis: a systematic review and Meta-analysis of randomized controlled trials. Am J Phys Med Rehabil. (2023) 102:292–9. doi: 10.1097/PHM.0000000000002088, PMID: 36111896

[15] Page MJ McKenzie JE Bossuyt PM Boutron I Hoffmann TC Mulrow CD . The PRISMA 2020 statement: an updated guideline for reporting systematic reviews. BMJ (Clin Res). (2021) 372:n71. doi: 10.1136/bmj.n71, PMID: 33782057 PMC8005924

[16] Higgins J. Cochrane handbook for systematic reviews of interventions version 6.4. London, United Kingdom. (2023).

[17] Altman RD. The classification of osteoarthritis. J Rheumatol Suppl. (1995) 43:42–3. 7752134

[18] D’Onofrio G Kirschner JS Prather HB Goldman D Rozanski A. Musculoskeletal exercise: its role in promoting health and longevity. Prog Cardiovasc Dis. (2023) 77, 25–36. doi: 10.1016/j.pcad.2023.02.006 36841491

[19] Herrmann SD Willis EA Ainsworth BE Barreira TV Hastert M Kracht CL . 2024 adult compendium of physical activities: a third update of the energy costs of human activities. J Sport Health Sci. (2024) 13:6–12. doi: 10.1016/j.jshs.2023.10.010, PMID: 38242596 PMC10818145

[20] Salanti G Del Giovane C Chaimani A Caldwell DM Higgins JP. Evaluating the quality of evidence from a network meta-analysis. PLoS One. (2014) 9:e99682. doi: 10.1371/journal.pone.0099682, PMID: 24992266 PMC4084629

[21] Lakens D. Calculating and reporting effect sizes to facilitate cumulative science: a practical primer for t-tests and ANOVAs. Front Psychol. (2013) 4:863. doi: 10.3389/fpsyg.2013.00863, PMID: 24324449 PMC3840331

[22] Bürkner P-C. Brms: an R package for Bayesian multilevel models using Stan. J Stat Softw. (2017) 80:1–28. doi: 10.18637/jss.v080.i01

[23] Williams DR Rast P Bürkner P-C Bayesian meta-analysis with weakly informative prior distributions. London, United Kingdom. (2018).

[24] Brooks SP Gelman A. General methods for monitoring convergence of iterative simulations. J Comput Graph Stat. (1998) 7:434–55.

[25] Inthout J Ioannidis JPA Borm GF Goeman JJ. Small studies are more heterogeneous than large ones: a meta-meta-analysis. J Clin Epidemiol. (2015) 68:860–9. doi: 10.1016/j.jclinepi.2015.03.017, PMID: 25959635

[26] Lin L Chu H Murad MH Hong C Qu Z Cole SR . Empirical comparison of publication Bias tests in Meta-analysis. J Gen Intern Med. (2018) 33:1260–7. doi: 10.1007/s11606-018-4425-7, PMID: 29663281 PMC6082203

[27] Cleophas TJ. Clinical trials: spline modeling is wonderful for nonlinear effects. Am J Ther. (2016) 23:e844–9. doi: 10.1097/MJT.0b013e318250f779, PMID: 23689089

[28] Wandel S Jüni P Tendal B Nüesch E Villiger PM Welton NJ . Effects of glucosamine, chondroitin, or placebo in patients with osteoarthritis of hip or knee: network meta-analysis. BMJ (Clin Res). (2010) 341:c4675. doi: 10.1136/bmj.c4675, PMID: 20847017 PMC2941572

[29] Hall M Hinman RS Wrigley TV Kasza J Lim BW Bennell KL. Knee extensor strength gains mediate symptom improvement in knee osteoarthritis: secondary analysis of a randomised controlled trial. Osteoarthr Cartil. (2018) 26:495–500. doi: 10.1016/j.joca.2018.01.018, PMID: 29427725

[30] Turkiewicz A Timpka S Thorlund JB Ageberg E Englund M. Knee extensor strength and body weight in adolescent men and the risk of knee osteoarthritis by middle age. Ann Rheum Dis. (2017) 76:1657–61. doi: 10.1136/annrheumdis-2016-210888, PMID: 28487313

[31] Stausholm MB Naterstad IF Alfredo PP Couppé C Fersum KV Leal-Junior ECP . Short- and long-term effectiveness of low-level laser therapy combined with strength training in knee osteoarthritis: a randomized placebo-controlled trial. J Clin Med. (2022) 11:3446. doi: 10.3390/jcm11123446, PMID: 35743513 PMC9225274

[32] Gong Z Li J He Z Li S Cao P Ruan G . Quadriceps strength is negatively associated with knee joint structural abnormalities-data from osteoarthritis initiative. BMC Musculoskelet Disord. (2022) 23:784. doi: 10.1186/s12891-022-05635-9, PMID: 35978313 PMC9382744

[33] Sadeghi A Rostami M Khanlari Z Zeraatchi A Jalili N Karimi Moghaddam A . Effectiveness of muscle strengthening exercises on the clinical outcomes of patients with knee osteoarthritis: a randomized four-arm controlled trial. Caspian J Intern Med. (2023) 14:433–42. doi: 10.22088/cjim.14.3.433, PMID: 37520861 PMC10379804

[34] Lo CN Wong N Ho S Ang EJH Leung BPL. Evaluating the effects of exercise on inflammation markers in musculoskeletal pain: a systematic review and meta-analysis. Sports (Basel, Switzerland). (2025) 13:168. doi: 10.3390/sports13060168, PMID: 40559680 PMC12197167

[35] Reis A Furtado GE Menuchi M Borges GF. The impact of exercise on interleukin-6 to counteract immunosenescence: methodological quality and overview of systematic reviews. Healthcare (Basel, Switzerland). (2024) 12:954. doi: 10.3390/healthcare12100954, PMID: 38786366 PMC11121001

[36] Sluka KA Frey-Law L Hoeger Bement M. Exercise-induced pain and analgesia? Underlying mechanisms and clinical translation. Pain. (2018) 159:S91–7. doi: 10.1097/j.pain.0000000000001235 30113953 PMC6097240

[37] Sharma L Dunlop DD Cahue S Song J Hayes KW. Quadriceps strength and osteoarthritis progression in malaligned and lax knees. Ann Intern Med. (2003) 138:613–9. doi: 10.7326/0003-4819-138-8-200304150-00006, PMID: 12693882

[38] Baker KR Nelson ME Felson DT Layne JE Sarno R Roubenoff R. The efficacy of home based progressive strength training in older adults with knee osteoarthritis: a randomized controlled trial. J Rheumatol. (2001) 28:1655–65. 11469475

[39] Hammami N Bouzouraa E Ölmez C Hattabi S Mhimdi N Khezami MA . Isokinetic knee strengthening impact on physical and functional performance, pain tolerance, and quality of life in overweight/obese women with patellofemoral pain syndrome. J Clin Med. (2024) 13:4696. doi: 10.3390/jcm13164696, PMID: 39200838 PMC11355345

[40] Wen CP Wu X. Stressing harms of physical inactivity to promote exercise. Lancet. (2012) 380:192–3. doi: 10.1016/S0140-6736(12)60954-4, PMID: 22818933

[41] Tonelli SM Rakel BA Cooper NA Angstom WL Sluka KA. Women with knee osteoarthritis have more pain and poorer function than men, but similar physical activity prior to total knee replacement. Biol Sex Differ. (2011) 2:12. doi: 10.1186/2042-6410-2-12, PMID: 22074728 PMC3228720

[42] Atasoy-Zeybek A Showel KK Nagelli CV Westendorf JJ Evans CH. The intersection of aging and estrogen in osteoarthritis. NPJ Womens Health. (2025) 3:15. doi: 10.1038/s44294-025-00063-1, PMID: 40017990 PMC11860234

[43] Gant H Ghimire N Min K Musa I Ashraf M Lawan A. Impact of the quadriceps angle on health and injury risk in female athletes. Int J Environ Res Public Health. (2024) 21:1547. doi: 10.3390/ijerph21121547, PMID: 39767389 PMC11675324

[44] Nold JI Fadai T Büchel C. Exercise-induced pain modulation is sex, opioid, and fitness-dependent and mediated by the medial frontal cortex. bioRxiv. (2024) 2024:600579. doi: 10.1101/2024.06.25.600579

[45] Aragon AA Tipton KD Schoenfeld BJ. Age-related muscle anabolic resistance: inevitable or preventable? Nutr Rev. (2023) 81:441–54. doi: 10.1093/nutrit/nuac062, PMID: 36018750

[46] Li X Li C Zhang W Wang Y Qian P Huang H. Inflammation and aging: signaling pathways and intervention therapies. Signal Transduct Target Ther. (2023) 8:239. doi: 10.1038/s41392-023-01502-8, PMID: 37291105 PMC10248351

