# Supplementary material for: Optimizing resistance training for pain management in knee and hip osteoarthritis: a pairwise and dose–response meta-analysis
Source: Front Public Health. 2025 Sep 2;13:1623679. doi: 10.3389/fpubh.2025.1623679 (PMC12436098; doi:10.3389/fpubh.2025.1623679)
Supplement: Supplementary file 1 [file Data_Sheet_1.DOCX]

**Optimizing Resistance Training for Pain Management in Knee and Hip Osteoarthritis: A Pairwise and Dose-Response Meta-Analysis**

Appendix

[1.Search strategy 2](#_Toc536)

[1.1 MEDLINE 2](#_Toc9075)

[1.2 Embase 3](#_Toc26703)

[1.3 Web of science 4](#_Toc1508)

[1.4 Cochrane Library 4](#_Toc29658)

[Appendix 2: Demographic characteristics of included studies 5](#_Toc29770)

[Appendix 3: Within-study risk of bias 16](#_Toc30901)

[Appendix 4: Publication bias assessments 17](#_Toc27698)

[Appendix 5: Convergence and posterior distributions 18](#_Toc6580)

[Appendix 6: R](#_Toc6832)^[2](#_Toc6832)^ [distribution 19](#_Toc6832)

[Appendix 7: Predicted responses 20](#_Toc22242)

# 1.Search strategy

## 1.1 MEDLINE

| **Search** | **Query** | **Results** |
| --- | --- | --- |
| #1 | (((((((((((Osteoarthritis, Hip[MeSH Terms]) OR (Hip Osteoarthritis[Transliterated Title])) OR (Osteoarthritis Of Hip[Transliterated Title])) OR (Osteoarthritis Of Hips[Transliterated Title])) OR (Coxarthrosis[Transliterated Title])) OR (Coxarthroses[Transliterated Title])) OR (Osteoarthritis of the Hip[Transliterated Title])) OR (Osteoarthritis, Knee[MeSH Terms])) OR (Knee Osteoarthritides[Title/Abstract])) OR (Knee Osteoarthritis[Title/Abstract])) OR (Osteoarthritis of Knee[Title/Abstract])) OR (Osteoarthritis of the Knee[Title/Abstract]) | 44965 |
| #2 | (((((((((((((((((((((((Resistance Training[MeSH Terms]) OR (Training, Resistance[Title/Abstract])) OR (Strength Training[Title/Abstract])) OR (Training, Strength[Title/Abstract])) OR (Weight-Lifting Strengthening Program[Title/Abstract])) OR (Strengthening Programs, Weight-Lifting[Title/Abstract])) OR (Strengthening Program, Weight-Lifting[Title/Abstract])) OR (Weight Lifting Strengthening Program[Title/Abstract])) OR (Weight-Lifting Strengthening Programs[Title/Abstract])) OR (Weight-Lifting Exercise Program[Title/Abstract])) OR (Exercise Programs, Weight-Lifting[Title/Abstract])) OR (Exercise Program, Weight-Lifting[Title/Abstract])) OR (Weight Lifting Exercise Program[Title/Abstract])) OR (Weight-Lifting Exercise Programs[Title/Abstract])) OR (Weight-Bearing Strengthening Program[Title/Abstract])) OR (Strengthening Programs, Weight-Bearing[Title/Abstract])) OR (Strengthening Program, Weight-Bearing[Title/Abstract])) OR (Weight Bearing Strengthening Program[Title/Abstract])) OR (Weight-Bearing Strengthening Programs[Title/Abstract])) OR (Weight-Bearing Exercise Program[Title/Abstract])) OR (Exercise Programs, Weight-Bearing[Title/Abstract])) OR (Exercise Program, Weight-Bearing[Title/Abstract])) OR (Weight Bearing Exercise Program[Title/Abstract])) OR (Weight-Bearing Exercise Programs[Title/Abstract]) | 19237 |
| #3 | ((Randomized controlled trial[Title/Abstract]) OR (Randomized[Title/Abstract])) OR (Placebo[Title/Abstract]) | 866478 |
| #4 | #1 AND #2 AND #3 | 488 |

## 1.2 Embase

| **Search** | **Query** | **Results** |
| --- | --- | --- |
| #1 | 'osteoarthritis, hip'/exp OR 'osteoarthritis, hip' OR 'osteoarthritis, knee'/exp OR 'osteoarthritis, knee' OR 'hip osteoarthritis':ab,ti OR 'osteoarthritis of hip':ab,ti OR 'osteoarthritis of hips':ab,ti OR 'coxarthrosis':ab,ti OR 'coxarthroses':ab,ti OR 'osteoarthritis of the hip':ab,ti OR 'knee osteoarthritides':ab,ti OR 'knee osteoarthritis':ab,ti OR 'osteoarthritis of knee':ab,ti OR 'osteoarthritis of the knee':ab,ti | 67028 |
| #2 | 'Resistance Training'/exp OR 'Training, Resistance':ab,ti OR 'Strength Training':ab,ti OR 'Training, Strength':ab,ti OR 'Weight-Lifting Strengthening Program':ab,ti OR 'Strengthening Programs, Weight-Lifting':ab,ti OR 'Strengthening Program, Weight-Lifting':ab,ti OR 'Weight Lifting Strengthening Program':ab,ti OR 'Weight-Lifting Strengthening Programs':ab,ti OR 'Weight-Lifting Exercise Program':ab,ti OR 'Exercise Programs, Weight-Lifting':ab,ti OR 'Exercise Program, Weight-Lifting':ab,ti OR 'Weight Lifting Exercise Program':ab,ti OR 'Weight-Lifting Exercise Programs':ab,ti OR 'Weight-Bearing Strengthening Program':ab,ti OR 'Strengthening Programs, Weight-Bearing':ab,ti OR 'Strengthening Program, Weight-Bearing':ab,ti OR 'Weight Bearing Strengthening Program':ab,ti OR 'Weight-Bearing Strengthening Programs':ab,ti OR 'Weight-Bearing Exercise Program':ab,ti OR 'Exercise Programs, Weight-Bearing':ab,ti OR 'Exercise Program, Weight-Bearing':ab,ti OR 'Weight Bearing Exercise Program':ab,ti OR 'Weight-Bearing Exercise Programs':ab,ti | 35427 |
| #3 | 'randomized controlled trial':ab,ti OR 'randomized':ab,ti OR 'placebo':ab,ti | 1234575 |
| #4 | #1 AND #2 AND #3 | 301 |
| #5 | #4 AND [embase]/lim NOT ([embase]/lim AND [medline]/lim) | 124 |

## 1.3 Web of science

| **Search** | **Query** | **Results** |
| --- | --- | --- |
| #1 | (TS=Osteoarthritis, Hip OR TS=Hip Osteoarthritis OR TS=Osteoarthritis Of Hip OR TS=Osteoarthritis Of Hips OR TS=Coxarthrosis OR TS=Coxarthroses OR TS=Osteoarthritis of the Hip OR TS=Osteoarthritis, Knee OR TS=Knee Osteoarthritides OR TS=Knee Osteoarthritis OR TS=Osteoarthritis of Knee OR TS=Osteoarthritis of the Knee) AND(TS=Resistance Training OR TS=Training, Resistance OR TS=Strength Training OR TS=Training, Strength OR TS=Weight-Lifting Strengthening Program OR TS=Strengthening Programs, Weight-Lifting OR TS=Strengthening Program, Weight-Lifting OR TS=Weight Lifting Strengthening Program OR TS=Weight-Lifting Strengthening Programs OR TS=Weight-Lifting Exercise Program OR TS=Exercise Programs, Weight-Lifting OR TS=Exercise Program, Weight-Lifting OR TS=Weight Lifting Exercise Program OR TS=Weight-Lifting Exercise Programs OR TS=Weight-Bearing Strengthening Program OR TS=Strengthening Programs, Weight-Bearing OR TS=Strengthening Program, Weight-Bearing OR TS=Weight Bearing Strengthening Program OR TS=Weight-Bearing Strengthening Programs OR TS=Weight-Bearing Exercise Program OR TS=Exercise Programs, Weight-Bearing OR TS=Exercise Program, Weight-Bearing OR TS=Weight Bearing Exercise Program OR TS=Weight-Bearing Exercise Programs) AND (TS=Randomized controlled trial OR TS=Randomized OR TS=Placebo) | 776 |

## 1.4 Cochrane Library

| **Search** | **Query** | **Results** |
| --- | --- | --- |
| #1 | MeSH descriptor: [Osteoarthritis, Hip] explode all trees | 1386 |
| #2 | MeSH descriptor: [Osteoarthritis, Knee] explode all trees | 7041 |
| #3 | (Hip Osteoarthritis OR Osteoarthritis Of Hip OR Osteoarthritis Of Hips OR Coxarthrosis OR Coxarthroses OR Osteoarthritis of the Hip OR Knee Osteoarthritides OR Knee Osteoarthritis OR Osteoarthritis of Knee OR Osteoarthritis of the Knee):ti,ab,kw | 21081 |
| #4 | MeSH descriptor: [Resistance Training] explode all trees | 5958 |
| #5 | (Training, Resistance OR Strength Training OR Training, Strength OR Weight-Lifting Strengthening Program OR Strengthening Programs, Weight-Lifting OR Strengthening Program, Weight-Lifting OR Weight Lifting Strengthening Program OR Weight-Lifting Strengthening Programs OR Weight-Lifting Exercise Program OR Exercise Programs, Weight-Lifting OR Exercise Program, Weight-Lifting OR Weight Lifting Exercise Program OR Weight-Lifting Exercise Programs OR Weight-Bearing Strengthening Program OR Strengthening Programs, Weight-Bearing OR Strengthening Program, Weight-Bearing OR Weight Bearing Strengthening Program OR Weight-Bearing Strengthening Programs OR Weight-Bearing Exercise Program OR Exercise Programs, Weight-Bearing OR Exercise Program, Weight-Bearing OR Weight Bearing Exercise Program OR Weight-Bearing Exercise Programs):ti,ab,kw | 32402 |
| #6 | (Randomized controlled trial OR Randomized OR Placebo):ti,ab,kw | 1348854 |
| #7 | #1 OR #2 OR #3 | 21081 |
| #8 | #4 OR #5 | 32402 |
| #9 | #6 AND #7 AND #8 | 803 |

**Appendix 2: Demographic characteristics of included studies**

| Country | Funded | OA_type | n | % Female | Mean Age (SD/Range) | Ill_duration (months) | Supervise | Treatment | Duration (weeks) | Intensity prescribed (METs) | Exercise dose (METs/week) | BMI | Timepoints available | Outcomes measured |
| --- | --- | --- | --- | --- | --- | --- | --- | --- | --- | --- | --- | --- | --- | --- |
| A Hermann2015 |  |  |  |  |  |  |  |  |  |  |  |  |  |  |
| Denmark | Y | H | 40 | 0.620 | 70.8 (7.5) | NA | NA | Usual care | 0 | 0 | 0 | 27.4 | Post | HOOS-pain |
| Denmark | Y | H | 40 | 0.680 | 70 (7.7) | NA | Y | Strength | 10 | 6 | 660 | 28.2 | Post | HOOS-pain |
| Adriano P Simão2012 |  |  |  |  |  |  |  |  |  |  |  |  |  |  |
| Brazil | N | K | 12 | 0.910 | 71 (5.3) | NA | NA | Keep daily | 0 | 0 | 0 | 26.7 | Post | WOMAC-pain |
| Brazil | N | K | 11 | 0.900 | 69 (3.7) | NA | Y | Strength | 12 | 5 | 750 | 29.8 | Post | WOMAC-pain |
| Ali Salli2010 |  |  |  |  |  |  |  |  |  |  |  |  |  |  |
| Turkey | N | K | 25 | 0.800 | 58.3 (6.67) | NA | NA | Keep daily | 0 | 0 | 0 | 32.82 | Post, 12 weeks | VAS-motion, VAS-rest |
| Turkey | N | K | 25 | 0.830 | 55.73 (8.23) | NA | Y | Strength | 8 | 5 | 720 | 31.5 | Post, 12 weeks | VAS-motion, VAS-rest |
| Turkey | N | K | 25 | 0.830 | 57.1 (6.75) | NA | Y | Strength | 8 | 5 | 720 | 32.65 | Post, 12 weeks | VAS-motion, VAS-rest |
| Aline Bassoli Gomiero2018 |  |  |  |  |  |  |  |  |  |  |  |  |  |  |
| Brazil | Y | K | 32 | 0.955 | 61.8 (6.4) | NA | NA | Educational | 0 | 0 | 0 | 23.6 | Post | SF-36-pain, VAS |
| Brazil | Y | K | 32 | 0.955 | 61.6 (6.8) | NA | NA | Educational | 0 | 0 | 0 | 24.1 | Post | SF-36-pain, VAS |
| Brazil | Y | K | 32 | 0.940 | 61.6 (6.8) | NA | Y | Strength | 16 | 4 | 385 | 24.1 | Post | VAS |
| Borges Jorge2014 |  |  |  |  |  |  |  |  |  |  |  |  |  |  |
| Brazil | Y | K | 31 | 1.000 | 59.9 (7.5) | 49.20 | NA | Waiting lists | 0 | 0 | 0 | 31.4 | Post | VAS, WOMAC-pain |
| Brazil | Y | K | 29 | 1.000 | 61.7 (6.4) | 58.80 | Y | Strength | 12 | 5 | 300 | 30.6 | Post | VAS, WOMAC-pain |
| Carina A Thorstensson2004 |  |  |  |  |  |  |  |  |  |  |  |  |  |  |
| Sweden | N | K | 31 | 0.520 | 57.3 (4.7) | NA | NA | Keep daily | 0 | 0 | 0 | 29.5 | Post, 18 weeks | KOOS-pain |
| Sweden | N | K | 30 | 0.500 | 54.8 (7.1) | NA | Y | Strength | 6 | 5 | 1000 | 29.6 | Post, 18 weeks | KOOS-pain |
| DA-HON LIN2009 |  |  |  |  |  |  |  |  |  |  |  |  |  |  |
| China | Y | K | 36 | 0.720 | 62.2 (6.7) | NA | NA | Keep daily | 0 | 0 | 0 | 24.66 | Post | WOMAC-pain |
| China | Y | K | 36 | 0.680 | 61.6 (7.2) | NA | Y | Strength | 8 | 4 | 810 | 23.74 | Post | WOMAC-pain |
| Georgios Evgeniadis2008 |  |  |  |  |  |  |  |  |  |  |  |  |  |  |
| Greece | N | K | 24 | 0.700 | 69.4 (1.9) | 148.44 | NA | Keep daily | 0 | 0 | 0 | 33.46 | Post | SF-36-pain |
| Greece | N | K | 24 | 0.850 | 67.1 (4.4) | 102.00 | Y | Strength | 8 | 4 | 472 | 34.73 | Post | SF-36-pain |
| Greece | N | K | 24 | 0.850 | 68.6 (5.9) | 116.28 | Y | Strength | 8 | 4 | 472 | 33.92 | Post | SF-36-pain |
| Gülbüz Samut2015 |  |  |  |  |  |  |  |  |  |  |  |  |  |  |
| Turkey | N | K | 13 | 1.000 | 60.92 (8.85) | NA | NA | Educational | 0 | 0 | 0 | 30.36 | Post | VAS, WOMAC-pain |
| Turkey | N | K | 15 | 1.000 | 62.46 (7.71) | NA | Y | Strength | 6 | 5 | 750 | 30.54 | Post | VAS, WOMAC-pain |
| Hakan2002 |  |  |  |  |  |  |  |  |  |  |  |  |  |  |
| Turkey | N | K | 6 | NA | 57 (9) | NA | NA | Keep daily | 0 | 0 | 0 | 32.2 | Post | NRS |
| Turkey | N | K | 9 | NA | 55 (12) | NA | Y | Strength | 8 | 4 | 720 | 31.6 | Post | NRS |
| Turkey | N | K | 9 | NA | 56 (12) | NA | Y | Strength | 8 | 4 | 720 | 32.1 | Post | NRS |
| Hongbo Chen2019 |  |  |  |  |  |  |  |  |  |  |  |  |  |  |
| China | N | K | 87 | 0.860 | 68.8 (6.96) | 72.00 | NA | Educational | 0 | 0 | 0 | 25.4 | Post | WOMAC-pain |
| China | N | K | 84 | 0.830 | 68.9 (7.78) | 80.40 | N | Strength | 12 | 5 | 600 | 25 | Post | WOMAC-pain |
| Levinger Pazit2018 |  |  |  |  |  |  |  |  |  |  |  |  |  |  |
| Australia | N | K | 10 | 0.560 | 70.44 (7.83) | NA | NA | Keep daily | 0 | 0 | 0 | 28.4 | Post | WOMAC-pain |
| Australia | N | K | 10 | 0.440 | 67.78 (6.28) | NA | Y | Strength | 8 | 7 | 700 | 28.2 | Post | WOMAC-pain |
| Luis Espejo Antúnez2012 |  |  |  |  |  |  |  |  |  |  |  |  |  |  |
| Spain | N | K | 14 | 0.640 | 82.21 (10.22) | NA | NA | Keep daily | 0 | 0 | 0 | na | Post | SF-36-pain, WOMAC-pain |
| Spain | N | K | 17 | 0.880 | 86.12 (4.96) | NA | Y | Strength | 4 | 4 | 350 | na | Post | SF-36-pain, WOMAC-pain |
| M Börjesson1996 |  |  |  |  |  |  |  |  |  |  |  |  |  |  |
| Sweden | Y | K | 34 | 0.500 | 64 (5) | 90.00 | NA | Keep daily | 0 | 0 | 0 | 27.7 | Post | Pain during walking |
| Sweden | Y | K | 34 | 0.500 | 64 (4) | 90.00 | Y | Strength | 5 | 4 | 420 | 28.4 | Post | Pain during walking |
| Matthew W Rogers2012 |  |  |  |  |  |  |  |  |  |  |  |  |  |  |
| USA | N | K | 8 | 0.670 | 71.2 (10.9) | NA | NA | Placebo | 0 | 0 | 0 | 30.8 | Post | WOMAC-pain |
| USA | N | K | 8 | 0.700 | 70.8 (6.5) | NA | N | Strength | 4 | 5 | 576 | 28.2 | Post | WOMAC-pain |
| USA | N | K | 8 | 0.700 | 70.8 (6.5) | NA | N | Strength | 8 | 5 | 576 | 28.2 | Post | WOMAC-pain |
| Mei-Hwa Jan2015 |  |  |  |  |  |  |  |  |  |  |  |  |  |  |
| China | N | K | 34 | 0.830 | 62.8 (6.3) | 42.00 | NA | Keep daily | 0 | 0 | 0 | 24.06 | Post | WOMAC-pain |
| China | N | K | 34 | 0.790 | 63.3 (6.6) | 39.60 | Y | Strength | 8 | 6 | 585 | 24.12 | Post | WOMAC-pain |
| China | N | K | 34 | 0.790 | 61.8 (7.1) | 33.60 | Y | Strength | 8 | 4 | 525 | 23.96 | Post | WOMAC-pain |
| Michael Wortley2013 |  |  |  |  |  |  |  |  |  |  |  |  |  |  |
| USA | Y | K | 9 | 0.670 | 70.5 (5) | NA | NA | Keep daily | 0 | 0 | 0 | 30 | Post | WOMAC-pain |
| USA | Y | K | 15 | 0.690 | 69.5 (6.7) | NA | Y | Strength | 10 | 5 | 600 | 30.5 | Post | WOMAC-pain |
| Ming-Cheng Weng2009 |  |  |  |  |  |  |  |  |  |  |  |  |  |  |
| China | Y | K | 33 | 0.800 | 64 (7.5) | NA | NA | Placebo | 0 | 0 | 0 | na | Post, 12 weeks | VAS |
| China | Y | K | 66 | 0.800 | 64 (7.5) | NA | Y | Strength | 8 | 5 | 495 | na | Post, 12 weeks | VAS |
| Paul DeVita2018 |  |  |  |  |  |  |  |  |  |  |  |  |  |  |
| USA | Y | K | 15 | 0.530 | 56.2 (8.9) | NA | NA | Keep daily | 0 | NA | 0 | 27.9 | Post | WOMAC-pain |
| USA | Y | K | 16 | 0.670 | 58.1 (6.5) | NA | Y | Strength | 12 | 5 | 882 | 26.4 | Post | WOMAC-pain |
| Riikka Juhakoski2011 |  |  |  |  |  |  |  |  |  |  |  |  |  |  |
| Finland | Y | H | 58 | 0.720 | 66.3 (6.6) | 102.00 | NA | Keep daily | 0 | 0 | 0 | 27.6 | Post, 24 weeks, 48 weeks, 72 weeks, 96 weeks | WOMAC-pain |
| Finland | Y | H | 60 | 0.680 | 66.9 (6.3) | 99.60 | Y | Strength | 12 | 4 | 158 | 27.48 | Post, 24 weeks, 48 weeks, 72 weeks, 96 weeks | WOMAC-pain |
| Robert A Bruce-Brand2012 |  |  |  |  |  |  |  |  |  |  |  |  |  |  |
| Ireland | N | K | 13 | 0.500 | 65.2 (3.1) | NA | NA | Usual care | 0 | 0 | 0 | 31.7 | Post, 6 weeks | WOMAC-pain |
| Ireland | Y | K | 13 | 0.500 | 65.2 (3.1) | NA | NA | Usual care | 0 | 0 | 0 | 31.7 | Post, 6 weeks | WOMAC-pain |
| Ireland | N | K | 14 | 0.400 | 63.4 (5.9) | NA | N | Strength | 6 | 4 | 315 | 33.9 | Post, 6 weeks | WOMAC-pain |
| Robert Topp2002 |  |  |  |  |  |  |  |  |  |  |  |  |  |  |
| USA | N | K | 35 | 0.800 | 60.94 (1.82) | NA | NA | Keep daily | 0 | 0 | 0 | na | Post | WOMAC-pain |
| USA | N | K | 35 | 0.710 | 65.57 (1.82) | NA | Y | Strength | 16 | 4 | 540 | na | Post | WOMAC-pain |
| USA | N | K | 32 | 0.660 | 63.53 (1.9) | NA | Y | Strength | 16 | 5 | 900 | na | Post | WOMAC-pain |
| Sang-arun Isaramalai2018 |  |  |  |  |  |  |  |  |  |  |  |  |  |  |
| Thailand | N | K | 45 | 0.800 | 63.7 (4.3) | NA | NA | Usual care | 0 | 0 | 0 | 24.7 | Post | WOMAC-pain |
| Thailand | N | K | 30 | 0.640 | 66.9 (4.5) | NA | N | Strength | 4 | 4 | 630 | 26.3 | Post | WOMAC-pain |
| Thailand | N | K | 30 | 0.640 | 66.9 (4.5) | NA | N | Strength | 8 | 4 | 630 | 26.3 | Post | WOMAC-pain |
| Thailand | N | K | 33 | 0.880 | 68 (5.8) | NA | N | Strength | 4 | 4 | 810 | 23.9 | Post | WOMAC-pain |
| Thailand | N | K | 33 | 0.880 | 68 (5.8) | NA | N | Strength | 8 | 4 | 810 | 23.9 | Post | WOMAC-pain |
| Schilke Joyce M1995 |  |  |  |  |  |  |  |  |  |  |  |  |  |  |
| USA | N | K | 10 | 0.740 | 68.4 (65) | 120.00 | NA | Keep daily | 0 | 0 | 0 | na | Post | OASI |
| USA | N | K | 10 | 0.740 | 64.5 (65) | 120.00 | Y | Strength | 8 | 4 | 315 | na | Post | OASI |
| Stephen P1997 |  |  |  |  |  |  |  |  |  |  |  |  |  |  |
| USA | N | K | 36 | 0.780 | 69.2 (6) | NA | NA | Keep daily | 0 | 0 | 0 | 32.5 | Post | The knee pain scale |
| USA | N | K | 34 | 0.680 | 67.2 (5.25) | NA | N | Strength | 72 | 4 | 525 | 30.1 | Post | The knee pain scale |
| STEPHEN P2012 |  |  |  |  |  |  |  |  |  |  |  |  |  |  |
| USA | Y | K | 15 | 0.730 | 68.4 (8.1) | NA | NA | Educational | 0 | 0 | 0 | 30.8 | Post | WOMAC-pain |
| USA | Y | K | 15 | 0.750 | 66.9 (4.9) | NA | Y | Strength | 12 | 5 | 825 | 28.4 | Post | WOMAC-pain |
| USA | Y | K | 15 | 0.800 | 65.9 (8.3) | NA | Y | Strength | 12 | 6 | 990 | 33.1 | Post | WOMAC-pain |
| Stephen P2021 |  |  |  |  |  |  |  |  |  |  |  |  |  |  |
| USA | Y | K | 124 | 0.390 | 64 (7) | NA | NA | Educational | 0 | 0 | 0 | 32 | Post | WOMAC-pain |
| USA | Y | K | 127 | 0.410 | 67 (9) | NA | Y | Strength | 72 | 6 | 1170 | 31 | Post | WOMAC-pain |
| USA | Y | K | 126 | 0.410 | 64 (8) | NA | Y | Strength | 72 | 4 | 630 | 31 | Post | WOMAC-pain |
| Tien-Wen Chen2014 |  |  |  |  |  |  |  |  |  |  |  |  |  |  |
| China | Y | K | 30 | NA | 63 (7.4) | NA | NA | Usual care | 0 | 0 | 0 | na | Post | Lequesne index, VAS |
| China | Y | K | 30 | NA | 63 (7.4) | NA | Y | Strength | 8 | 5 | 751 | na | Post | Lequesne index, VAS |

#

# Appendix 3: Within-study risk of bias


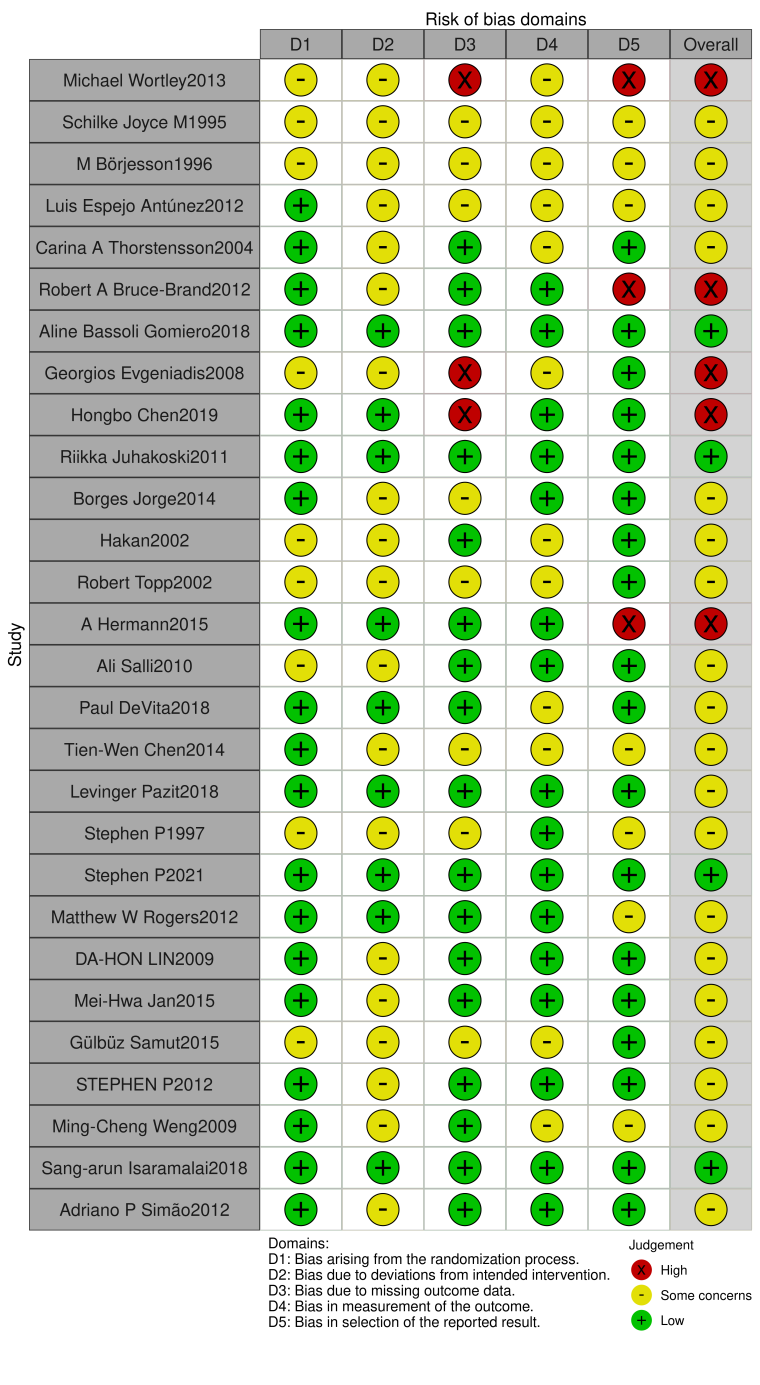


**Figure S1. The details of risk of bias**

**
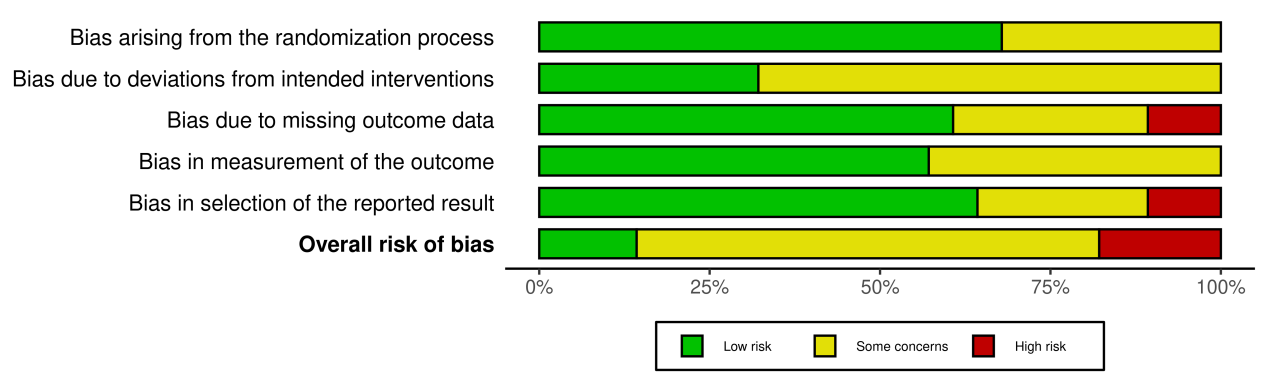
**

**Figure S2. The summary of risk of bias**

# Appendix 4: Publication bias assessments


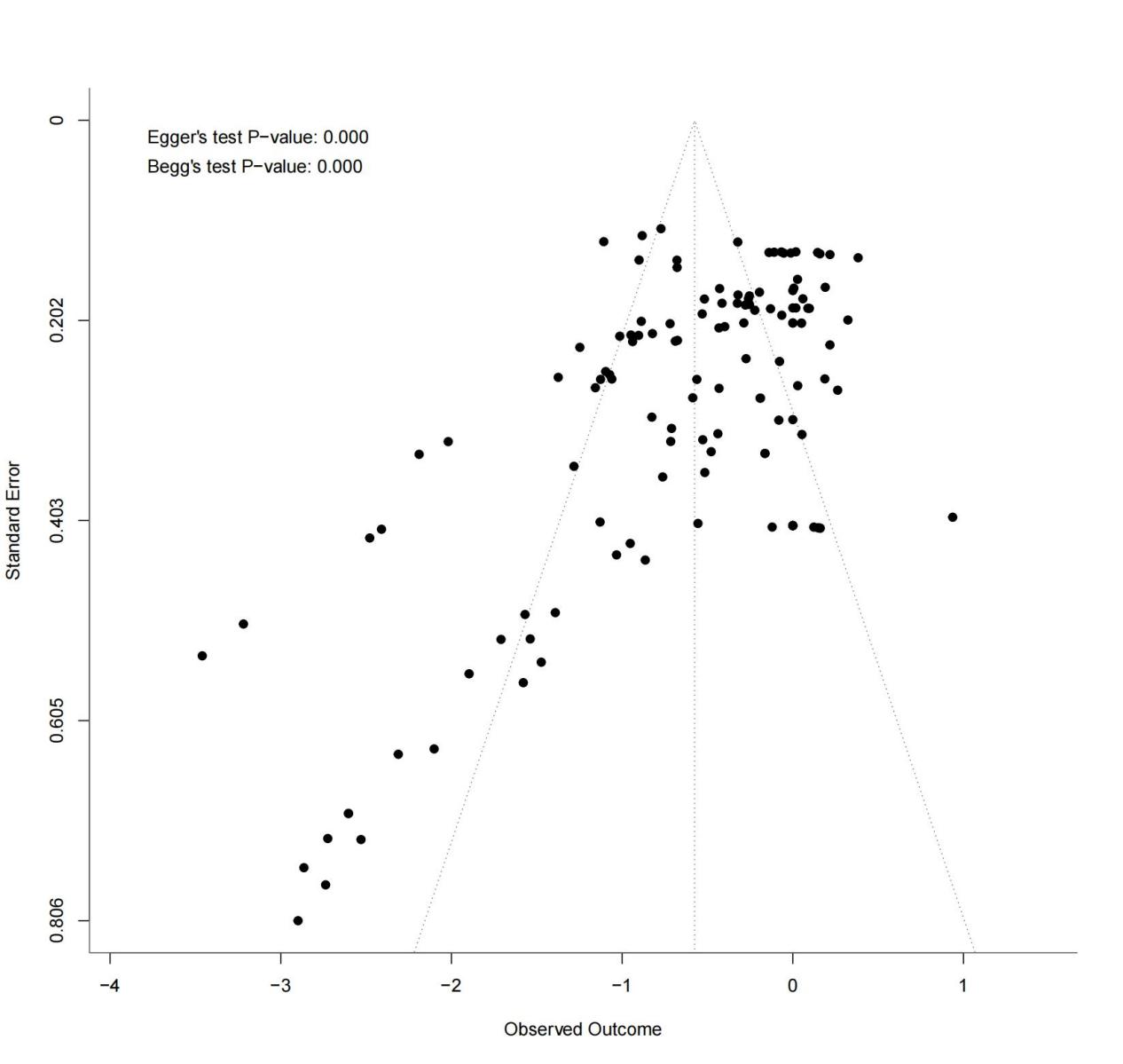


**Figure S3. Funnel plot**

# Appendix 5: Convergence and posterior distributions


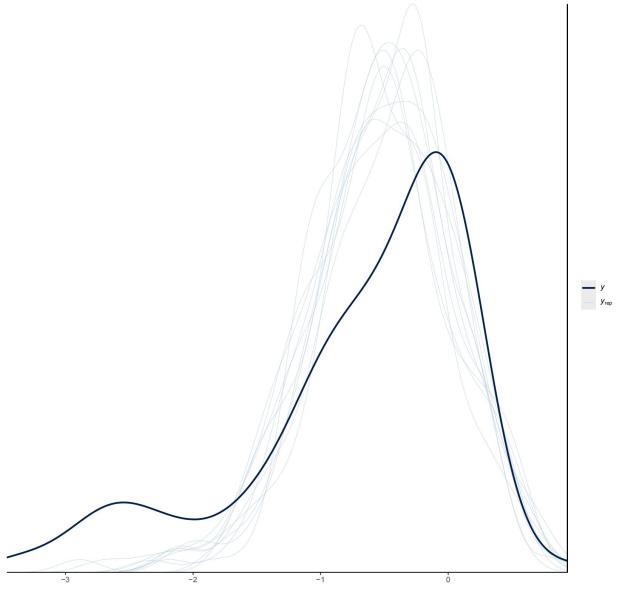


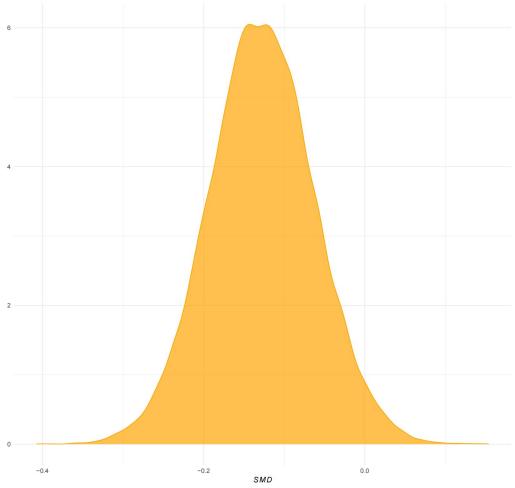

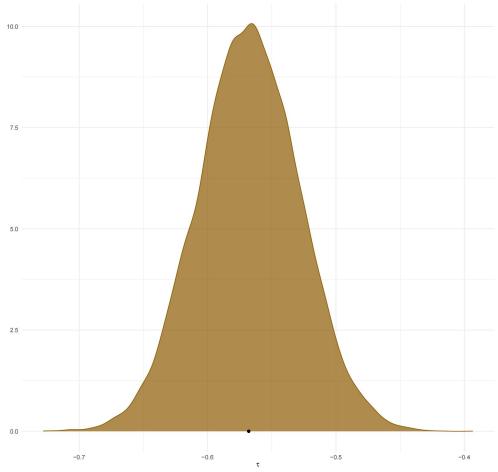


**Figure S4. Convergence and posterior distributions**

# Appendix 6: R^2^ distribution


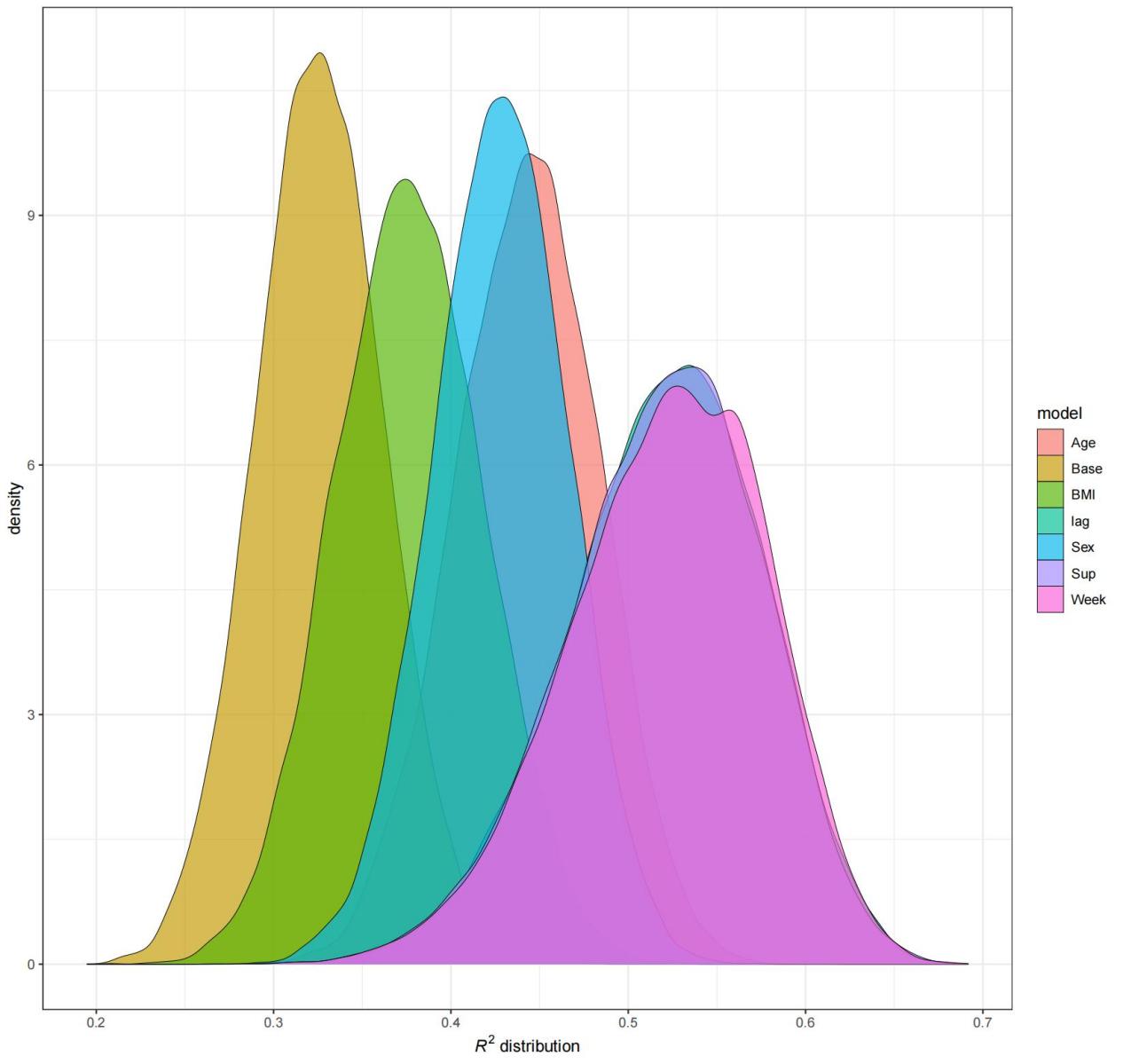


**Figure S5.** R^2^ distribution for all moderating factors

# Appendix 7: Predicted responses

**Table S1. Predicted responses of resistance training for pain.**

| **Weekly_dose** | **Pred** | **Se** | **Lower** | **Upper** |
| --- | --- | --- | --- | --- |
| 0 | 0.1053 | 0.49407 | -0.70738 | 0.91798 |
| 20 | 0.0716 | 0.47062 | -0.7025 | 0.84571 |
| 40 | 0.0379 | 0.44726 | -0.69778 | 0.77359 |
| 60 | 0.0042 | 0.42401 | -0.69323 | 0.70164 |
| 80 | -0.0295 | 0.40089 | -0.68889 | 0.6299 |
| 100 | -0.0632 | 0.37791 | -0.6848 | 0.55841 |
| 120 | -0.09689 | 0.35511 | -0.68099 | 0.4872 |
| 140 | -0.13059 | 0.33252 | -0.67754 | 0.41635 |
| 160 | -0.16429 | 0.31019 | -0.67451 | 0.34593 |
| 180 | -0.19797 | 0.28821 | -0.67203 | 0.27609 |
| 200 | -0.23154 | 0.26671 | -0.67023 | 0.20716 |
| 220 | -0.2649 | 0.24586 | -0.66931 | 0.13951 |
| 240 | -0.29796 | 0.22584 | -0.66944 | 0.07352 |
| 260 | -0.33062 | 0.20684 | -0.67084 | 0.00959 |
| 280 | -0.36279 | 0.18903 | -0.67371 | -0.05187 |
| 300 | -0.39436 | 0.17261 | -0.67828 | -0.11045 |
| 320 | -0.42525 | 0.15777 | -0.68476 | -0.16574 |
| 340 | -0.45532 | 0.14469 | -0.69332 | -0.21732 |
| 360 | -0.48446 | 0.13352 | -0.70408 | -0.26485 |
| 380 | -0.51254 | 0.12431 | -0.71701 | -0.30807 |
| 400 | -0.53941 | 0.11704 | -0.73193 | -0.3469 |
| 420 | -0.56497 | 0.11158 | -0.7485 | -0.38143 |
| 440 | -0.58908 | 0.10769 | -0.76621 | -0.41194 |
| 460 | -0.61162 | 0.10507 | -0.78445 | -0.43879 |
| 480 | -0.6325 | 0.10339 | -0.80257 | -0.46243 |
| 500 | -0.6516 | 0.10235 | -0.81994 | -0.48325 |
| 520 | -0.66883 | 0.10169 | -0.8361 | -0.50156 |
| 540 | -0.68412 | 0.10126 | -0.85068 | -0.51756 |
| 560 | -0.69742 | 0.10099 | -0.86353 | -0.53132 |
| 580 | -0.70869 | 0.10087 | -0.8746 | -0.54278 |
| 600 | -0.7179 | 0.10096 | -0.88397 | -0.55184 |
| 620 | -0.72506 | 0.10136 | -0.89178 | -0.55835 |
| 640 | -0.73019 | 0.10215 | -0.89821 | -0.56217 |
| 660 | -0.73333 | 0.10341 | -0.90342 | -0.56324 |
| 680 | -0.73452 | 0.10514 | -0.90746 | -0.56158 |
| 700 | -0.73382 | 0.10732 | -0.91034 | -0.55729 |
| 720 | -0.73128 | 0.10985 | -0.91197 | -0.5506 |
| 740 | -0.72699 | 0.11259 | -0.91218 | -0.54179 |
| 760 | -0.72101 | 0.1154 | -0.91082 | -0.5312 |
| 780 | -0.71344 | 0.11811 | -0.90772 | -0.51916 |
| 800 | -0.70437 | 0.1206 | -0.90275 | -0.50599 |
| 820 | -0.6939 | 0.12275 | -0.89581 | -0.49198 |
| 840 | -0.68212 | 0.12448 | -0.88687 | -0.47736 |
| 860 | -0.66912 | 0.12575 | -0.87596 | -0.46229 |
| 880 | -0.65502 | 0.12656 | -0.86319 | -0.44685 |
| 900 | -0.6399 | 0.12696 | -0.84872 | -0.43107 |
| 920 | -0.62386 | 0.12703 | -0.8328 | -0.41491 |
| 940 | -0.60699 | 0.12691 | -0.81574 | -0.39824 |
| 960 | -0.58937 | 0.12677 | -0.79789 | -0.38085 |
| 980 | -0.5711 | 0.12679 | -0.77964 | -0.36255 |
| 1000 | -0.55226 | 0.12716 | -0.76142 | -0.3431 |
| 1020 | -0.53294 | 0.12809 | -0.74362 | -0.32225 |
| 1040 | -0.51318 | 0.12975 | -0.7266 | -0.29977 |
| 1060 | -0.49306 | 0.1323 | -0.71067 | -0.27544 |
| 1080 | -0.47262 | 0.13584 | -0.69606 | -0.24917 |
| 1100 | -0.45192 | 0.14043 | -0.68291 | -0.22093 |
| 1120 | -0.43103 | 0.14606 | -0.67127 | -0.19078 |
| 1140 | -0.40999 | 0.15266 | -0.6611 | -0.15888 |
| 1160 | -0.38887 | 0.16016 | -0.6523 | -0.12543 |
| 1180 | -0.36771 | 0.16841 | -0.64473 | -0.0907 |
| 1200 | -0.34656 | 0.17731 | -0.63821 | -0.0549 |
| 1220 | -0.3254 | 0.18677 | -0.6326 | -0.0182 |
| 1240 | -0.30425 | 0.19669 | -0.62777 | 0.01928 |
| 1260 | -0.28309 | 0.20702 | -0.6236 | 0.05742 |
| 1280 | -0.26194 | 0.21769 | -0.62001 | 0.09613 |
| 1300 | -0.24078 | 0.22866 | -0.6169 | 0.13534 |
| 1320 | -0.21963 | 0.23989 | -0.61422 | 0.17496 |
| 1340 | -0.19847 | 0.25135 | -0.6119 | 0.21496 |
| 1360 | -0.17732 | 0.26299 | -0.6099 | 0.25527 |
| 1380 | -0.15616 | 0.27481 | -0.60818 | 0.29586 |
| 1400 | -0.13501 | 0.28677 | -0.60671 | 0.33669 |
| 1420 | -0.11385 | 0.29887 | -0.60545 | 0.37774 |
| 1440 | -0.0927 | 0.31108 | -0.60437 | 0.41898 |
| 1460 | -0.07154 | 0.32339 | -0.60347 | 0.46038 |
| 1480 | -0.05039 | 0.33579 | -0.60271 | 0.50194 |
| 1500 | -0.02923 | 0.34827 | -0.60209 | 0.54363 |
